# Bayesian multilevel model of micro RNA levels in ovarian-cancer and healthy subjects

**DOI:** 10.1371/journal.pone.0221764

**Published:** 2019-08-29

**Authors:** Paweł Wiczling, Emilia Daghir-Wojtkowiak, Roman Kaliszan, Michał Jan Markuszewski, Janusz Limon, Magdalena Koczkowska, Maciej Stukan, Alina Kuźniacka, Magdalena Ratajska

**Affiliations:** 1 Department of Biopharmaceutics and Pharmacodynamics, Medical University of Gdańsk, Gen. J. Hallera, Gdańsk, Poland; 2 Department of Biology and Genetics, Medical University of Gdańsk, Dębinki, Gdańsk, Poland; 3 Department of Gynecological Oncology, Gdynia Oncology Centre, Powstania Styczniowego, Gdynia, Poland; Institut de Pharmacologie Moleculaire et Cellulaire, FRANCE

## Abstract

In transcriptomics, micro RNAs (miRNAs) has gained much interest especially as potential disease indicators. However, apart from holding a great promise related to their clinical application, a lot of inconsistent results have been published. Our aim was to compare the miRNA expression levels in ovarian cancer and healthy subjects using the Bayesian multilevel model and to assess their potential usefulness in diagnosis. We have analyzed a case-control observational data on expression profiling of 49 preselected miRNA-based ovarian cancer indicators in 119 controls and 59 patients. A Bayesian multilevel model was used to characterize the effect of disease on miRNA levels controlling for differences in age and body weight. The difference between the miRNA level and health status of the patient on the scale of the data variability were discussed in the context of their potential usefulness in diagnosis. Additionally, the cross-validated area under the ROC curve (AUC) was used to assess the expected out-of-sample discrimination index of a different sets of miRNAs. The proposed model allowed us to describe the set of miRNA levels in patients and controls. Three highly correlated miRNAs: miR-101-3p, miR-142-5p, miR-148a-3p rank the highest with almost identical effect sizes that ranges from 0.45 to 1.0. For those miRNAs the credible interval for AUC ranged from 0.63 to 0.67 indicating their limited discrimination potential. A little benefit in adding information from other miRNAs was observed. There were several miRNAs in the dataset (miR-604, hsa-miR-221-5p) for which inferences were uncertain. For those miRNAs more experimental effort is needed to fully assess their effect in the context of new hits discovery and usefulness as disease indicators. The proposed multilevel Bayesian model can be used to characterize the panel of miRNA profile and to assess the difference in expression levels between healthy and cancer individuals.

## Introduction

MicroRNAs (miRNAs) are abundant classes of endogenous, small non-coding RNAs of 17–25 nucleotides in length generated from 70–100 nucleotides-long hairpin precursors, which regulate gene expression post-transcriptionally by affecting the translation of target messenger RNAs (mRNAs) [1]. mRNA target recognition by a single miRNA is found in different regions of mRNA, particularly in the 3’ untranslated region (3’UTR), 5’ untranslated region (5’UTR) and in the coding sequences [2], depending solely on a complementarity with the 6–8 5′ nucleotides of the miRNAs. The same miRNA may have different effects on the same disease. A single miRNA can affect hundreds of mRNA targets acting as oncogenes or tumor suppressors in a cellular-dependent context and depending on the genes targeted [3, 4]. Accumulated evidences have shown that miRNA expression is altered in most types of cancer being involved in a regulation of a wide range of developmental, physiological and cellular processes e.g. proliferation, adhesion, apoptosis and angiogenesis [5].

Therefore, a lot of effort has been paid towards searching for promising miRNA hits for diagnosis and treatment of various types of cancer e.g. breast cancer [6], leukemia [7,8], liver cancer [9,10], ovarian cancer [11], pancreatic and prostate cancer [12,13], and other diseases as well (cardiovascular, metabolic diseases, neurodegenerative disorders) [14,15,16].

Traditional experiments towards searching for novel miRNA-disease associations cost a lot of manpower, material and financial resources. For this reason, much effort is undertaken towards building effective and accurate computational models to reveal the potential relationship between disease and miRNA according to the hypothesis that miRNAs with similar functions are likely to be involved in diseases with similar phenotypes and vice versa (Bandyopadhyay, et al., 2010).

According to a state-of-the-art of existing miRNA-disease association studies, computational prediction models have been divided into four categories, (i) score function-based, (ii) complex network algorithm-based, (iii) machine learning-based, and (iv) multiple biological information-based models (comprehensively described in the review by Chen et al. [17].

Briefly and generally, the score function-based models assume that there is higher probability of association between functional-related miRNAs and phenotypically similar diseases. As its foundations lie in the probabilistic theory, assumption of prior knowledge on data distribution may affect prediction especially if the data informational content is poor. However, due to the lack of experimentally supported miRNA–target interactions, score function-based models provide high rates of false-positive and false-negative results. In this family of models, the most up-to-date models is The Within and Between Score for MiRNA–Disease Association prediction (WBSMDA) [18].

The complex network algorithm-based methods involve different aspects of miRNA similarity networks and disease similarity networks. This method is based on the use of topological information of the miRNA-disease bilayer network assuming that functionally similar miRNAs are more likely to be involved in a similar disease and vice versa which is in accordance with biological experiments. However, the drawback of this methods lies in a difficulty in their application to a new disease unless more experimental data on miRNA/disease function interaction network is collected. One of the most up-to date examples of this algorithms are: random walk-based computational model of Random Walk with Restart for MiRNA–Disease Association (RWRMDA) [19], random walk on the miRNA–disease bilayer network (MIDP) [20], Path-Based computational model for MiRNA–Disease Association (PBMDA) [21], Heterogeneous Graph Inference for MiRNA–Disease Association prediction (HGIMDA) [22], Random Walk and Binary Regression-based MiRNA-Disease Association prediction (RWBRMDA) [23].

The machine learning-based prediction models use machine learning algorithms for predictions via extracting the most relevant features or solving specific optimization problems. These kinds of methods predict the potential miRNAs for a new disease, without any previous associated disease. Machine learning-based model can incorporate different covariates for the final prediction offering improvement in the prediction performance. The most up-to-date examples of such algorithms are KRLSM for predicting miRNA–disease associations using Kronecker RLS based on heterogeneous omics data [24], Matrix Completion for MiRNA–Disease Association prediction model (MCMDA) [25], Ranking based k-nearest-neighbors for MiRNA–Disease Association prediction (RKNNMDA) [26], Adaptive Boosting for MiRNA-Disease Association prediction (ABMDA) [27], Negative Samples Extraction based MiRNA-Disease Association prediction (NSEMDA) [28]. Multiple biological information-based models assume integration of information between miRNA–gene and disease–protein associations to explore miRNA-related and disease-related associations. The most up-to-date examples of such algorithms are computational model to infer miRNA–Protein–Disease associations (miRPD) [29] and computational framework named KBMFMDI [30], Adaptive Multi-View Multi-Label learning(AMVML) [31], Matrix Decomposition and Heterogeneous Graph Inference for miRNA-disease association prediction (MDHGI) [32].

The above-mentioned computational-based methods have their own strengths and weaknesses. The latter may result from: (1) rare existence of identified miRNA–disease associations; (2) unavailable data on negative miRNA–disease associations; (3) limited biological data sets about miRNAs; (4) difficulties in applying computational models to miRNAs without any prior knowledge on associated diseases [17].

At present, as a complement to existing computer-based methods, more interest is paid to those based on Bayesian statistics i.e. neoteric Bayesian model (KBMFMDA) which combines kernel-based nonlinear dimensionality reduction, matrix factorization and binary classification [33] and Bayesian probabilistic matrix factorization (MDBPMF), in order to discover novel miRNA-disease associations [34], or variational Bayesian Gaussian mixture model (VB-GMM) to predict miRNA target genes [35].

The general idea of all computational models is to find the candidate miRNAs potentially associated with the disease of interest and further confirm these top miRNAs in experiments. As mentioned earlier, the pros of this approach lie in saving a lot of experimental effort with respect to miRNA-disease association.

Apart from computational models which assess miRNA-disease associations, a different aspect of analysing miRNA data from meta analyses or a single experiment involves estimation with quantified uncertainty based on effect size and credible intervals. This approach was used by Eftekharian et al. [36] and Sayad et al. [37] and is attributed to multilevel Bayesian models. This approach represents the idea that data generated in experiments can be described via mathematical models. The concept behind fitting a model to the data lies in generating thousands of random samples of the actually-observed data in order to estimate the values of model parameters with appropriate uncertainty. The more data with greater informational content, the better precision of estimation (and otherwise, less data with lesser information content, worse precision of estimation). Bayesian multilevel models encourage shifting to estimation with uncertainty and magnitude of uncertainty aiming at estimating precision rather than testing hypotheses promoting black-and-white thinking. [38]. Such distinction between null hypothesis significance testing (NHST), on the one hand, and estimation with quantified uncertainty on the other, has indirectly been promoted by the presence of “noise” in the data. For this reason, the effect size statistics seems to be a useful measure providing the information on (i) the magnitude or strength of outcomes, (ii) power for future studies and may be useful in meta-analyses while summarizing the effect sizes across independent studies [39]. To estimate the effect size for ANOVA models, the following statistics are usually used: (i) eta-squared (*ƞ*^*2*^), (ii) partial eta-sqaured (*ƞ*_*p*_^*2*^) and (iii) omega-squared (*ɷ*^*2*^). Moreover, to simply judge on the mean differences between two measurements one can calculate: R^2^ if one performs regression and evaluates the correlation between 2 variables, or Cohen’s *d* if one performs a t-test and want to know mean differences in a t-test [40].

Therefore, to assess the relationship between the presence of ovarian cancer and miRNA expression and to judge on the importance of the effect of disease on miRNAs concluding how certain their magnitude can be estimated, we develop a data-driven multilevel Bayesian model. The model included correlations between miRNAs and accounted for inter-individual and assay variability. We also discuss the obtained results in the context of traditional (Frequentists) approach based on controlling the false discovery rate (FDR).

## Material and methods

This study was approved by the Research Ethics Committee of the Medical University of Gdansk (NKBBN/399/2011-2012). Written informed consent was obtained from all individual participants included in the study.

### Structure of the dataset

Dataset used in this study consisted of vector Y of size N = 8722 (number of observations, n = 1 … N) representing centered and standardized miRNA levels measured in plasma and transformed to a natural logarithmic (log) scale for *K* = 49 (*k* = 1 … *K*) different miRNAs determined in *I* = 178 individuals (*i = 1…I*, 119 controls and 59 patients) under two replicates. Y is related to the measured quantification cycle, *CT*, through the standard equation (40 –CT) log(2). A control miRNA (UniSp6) constituted cDNA synthesis control and was not included in the data analysis as it was used as an internal control of miRNA profiling. A set of vectors was used to denote indexes representing study design with *k[n]* denoting an indicator for miRNA and *i[n]* denoting indicator for a subject. Health status (0 corresponding to control and 1 to patients) constituted the available discrete covariate and was denoted as *I* x 1 vector DIS. Two continuous covariates were available: age denoted as *I* x 1 vector AGE and body weight denoted as *I* x 1 vector BW. The mean and standard deviation (SD) of data prior to centering and standardization equaled 4.03 and 1.93. Raw data are in S1 Data. The details on the experimental procedure regarding miRNA expression profiling can be found in the S1 Appendix.

### Model development

The following multilevel model was used to describe the miRNA data:

$$y_{n}∼N(\mu_{k[n]}+\beta_{DIS,k[n]}{DIS}_{i[n]}+\beta_{BW,k[n]}{BW}_{i[n]}+\beta_{AGE,k[n]}{AGE}_{i[n]}+\eta_{i[n],k[n]},\sigma_{k[n]})$$ (1)

$$\eta_{i,1\ldots K}∼MVN(0,\Omega )$$ (2)

where *N* and *MVN* denote the normal and multivariate normal distribution, a tilde (~) denotes "has the probability distribution of", i.e. the values of *y*_*n*_ and *η*_*i*,1…*K*_ are randomly drawn from the given (normal and multivariate normal) distribution, *y*_*n*_ represents the dependent variable; *μ*_*k*_ is the typical miRNA level in a healthy subject of age 52.7 years and body weight of 67.3 kg, *β*_*DIS*,*k*_ describes the effect of disease for a particular miRNA*; β*_*AGE*,*k*_ and *β*_*BW*,*k*_ correspond to the effect of age and body weight covariates on miRNA levels, *σ*_*k*_ denotes standard deviation associated with measurement error for *k*^*th*^ miRNA. *η*_*i*.1…*K*_ is the between-subject variability of 49^th^ miRNA that was modeled using a MVN distribution with covariance matrix *Ω*.

The single missing value of a disease status was modeled assuming Bernoulli distribution parametrized using the proportion of cancer/healthy subjects in the data set.

$${DIS}_{i}∼Bern(0.33)$$ (3)

Similarly, the missing values for AGE and BW were assumed to be normally distributed with mean zero and standard deviation equal to 1 (thus to be approximately in a range of ages and body weight of subjects included in the study).

$${AGE}_{i},{BW}_{i}∼N(0,1)$$ (4)

The following prior distribution was assumed during model building process:

$$\sigma_{k}∼N(0,1)T(0,)$$ (5)

$$\mu_{k}∼N(0,5)$$ (6)

$$\beta_{DIS,k},\beta_{BW,k},\beta_{AGE,k}∼N(0,1)$$ (7)

For the standard deviation, half-normal distribution (expressed as T(0,) in Eq 4) ensuring positive values was used (Eq 5). We also assumed the normal distribution with mean zero and standard deviation of 5 for the mean level of miRNAs (Eq 6). A scaled inverse-Wishart prior was used for the variance-covariance matrix. This was necessary as it allows to estimate the scale parameters and the correlations from the hierarchical data. To implement it we expanded Ω to:

Ω=diag(ζ)Qdiag(ζ) (8)

where *Q* is the unscaled covariance matrix being given the inverse-Wishart model and *ζ*_*k*_ is a scaling factor being given a half-normal model for each miRNA:

$$Q∼{Inv-Whishart}_{K+1}(I_{K})$$ (9)

$$\zeta_{k}∼N(0,1)T(0,)$$ (10)

where *I*_*K*_ is a scale, here K x K identity matrix and K+1 denotes degrees of freedom. The *ζ* and Q parameters cannot be interpreted separately, but allow to calculate the covariance matrix Ω = *diag*(*ω*)*ρ diag*(*ω*), and the most interesting quantities, like standard deviations and correlation matrix [41]:

$$\omega_{k}=\sqrt{\Omega_{kk}}={|\zeta }_{k}|\sqrt{Q_{kk}}$$ (11)

$$\rho_{kk\prime }=\zeta_{k}\zeta_{k^{\prime }}Q_{kk^{\prime }}/{(\omega }_{k}\omega_{k\prime })$$ (12)

To illustrate the magnitude of the difference between patients and the control group we calculated the effect size for each miRNA on the scale of data variability (*d*_*k*_).

$$d_{k}=\beta_{DIS,k}/\sqrt{\omega_{k}^{2}+\sigma_{k}^{2}}$$ (13)

The effect size along with the associated uncertainty is a useful measure to assess the potential diagnostic value of a single miRNA. The values of *d*_*k*_ larger than 1.5 indicate that the miRNA levels in cancer and patient subjects differ considerably (the underlying normal distributions are almost baseline separated).

### AUC under the ROC

To assess the expected out of sample discrimination potential of a subset of miRNA, AUC under the ROC curve was calculated using 10-fold cross-validation. For that purpose the patients from the original data were randomly partitioned into 10 subsamples. Out of the 10 subsamples, a single subsample was excluded from the analysis. The remaining 9 subsamples were used to calculate the probability of cancer for each excluded subject (*p*_*i*_). The cross-validation process was then repeated 10 times, with each of the 10 subsamples used exactly once as the validation data. The results from the folds were combined and summarized as AUC under the ROC curve. The probability of having cancer for a particular individual was calculated transforming the proposed linear model to its logistic representation [42]:

$$logit(p_{i})=\log\left(0.333\right)-0.5(2\mu_{k∊j}+\beta_{DIS,k∊j})\Sigma_{k∊j}^{-1}\beta_{\beta_{DIS,k∊j}}^{T}+{y_{k∊j}\Sigma }_{k∊j}^{-1}\beta_{\beta_{DIS,k∊j}}^{T}$$ (14)

where *j* denote a subset of miRNA used for predictions, *y*_*j*_ denote miRNA observations from one repetition only, *Σ* is a sum of the inter-individual and residual variability *Σ* = *Ω*+*diag*(*σ*^2^); log(0.33) denotes the ratio of prior probabilities here assumed equal to the proportion of cancer/healthy subjects in the data.

### Model assessment

For model diagnostic purposes we plotted (i) weighted residuals versus miRNA and (ii) weighted residuals versus fitted values. This graph evaluated the variability of the observations across each miRNA and assessed the presence of a pattern or trend in the residuals. The weighted residuals should be distributed across zero line with standard deviation near one. If miRNA observations falls outside this range, it indicates model misspecification.

### False discovery rate approach

The FDR method was adopted by ranking the raw *p* values from the lowest to the highest, multiplying each *p* value by the number of variables, and dividing by its rank order. If the FDR-corrected *p-*value is less than the significance level 0.05 a variable is conventionally labelled statistically significant.

### Technical

The model was developed using JAGS 4.0.0. with *rjags*, *runjags* and *coda* packages in R environment. Three MCMC chains of 100000 iterations were simulated. The first 1000 iterations of each chain were discarded and every 3rd sample was retained. Thus 1000 MCMC samples were used for subsequent analyses. Model convergence was assessed by Gelman-Rubin diagnostics available in JAGS. The MCMC chains were assumed to have reached the stationary distribution if Gelman-Rubin values were less than 1.2 for all parameters. Furthermore, the trace history of MCMC samples for all chains were examined visually for all parameters, for which ‘fuzzy caterpillar’ suggests that MCMC chains had reached a stationary distribution. The code for the model is available in the S1 Appendix. The FDR was calculated given a set of *p*-values adjusted using Benjamini & Hochberg method with *stats* package in R environment.

## Results

### Biological concept of the study

The whole study design initialized with the determination of 752 miRNA levels in 59 samples (first stage of the study): (i) control (n = 16), (ii) ovarian cancer with no BRCA1/2 mutation (-/-) (n = 33) and (iii) ovarian cancer with BRCA1 or BRCA2 mutation (+/+) (n = 10). Further (second stage of the study), based on concentration differences reported between patients and controls in the first stage (based on *p*-value with FDR correction) and the available literature reports, 49 miRNAs were selected out of 752 hits and further measured in 178 individuals: (i) control (n = 118), (ii) ovarian cancer with no BRCA1/2 mutation (-/-) (n = 49) and (iii) ovarian cancer with BRCA1 or BRCA2 mutation (+/+) (n = 10). The second stage of the study included a separate group of individuals not included in the first stage of the study.

### Dataset characteristics

**Fig 1:**
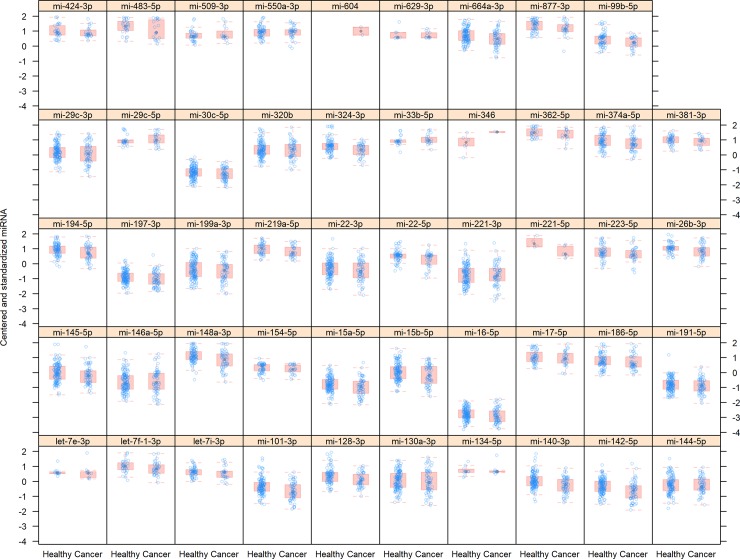
Raw data (centered and standardized miRNA levels) summarized as boxplots for 49 miRNAs in patients and controls. The box and whiskers plots depict mean, 25th and 75th percentiles. Blue dots overlaid are individual data points.

The raw data used in this study covered 49 miRNAs measured in 178 individuals (59 ovarian cancer patients and 119 controls) (Fig 1). Detailed characteristics of the available covariates is presented in Table 1. The mean for age and weight of individuals was 52.6 (±13.7) and 67.2 (±11.6).

**Table 1:** Demographic characteristic of subjects included in the study.

	All subjects	Percent of missing data
**Health status**	-	0.56%
**Age, mean (±SD)**	52.67 (±13.7)	2.2%
**Weight, mean (±SD)**	67.29 (±11.66)	36.5%

### Effect of health status on miRNA levels

**Fig 2:**
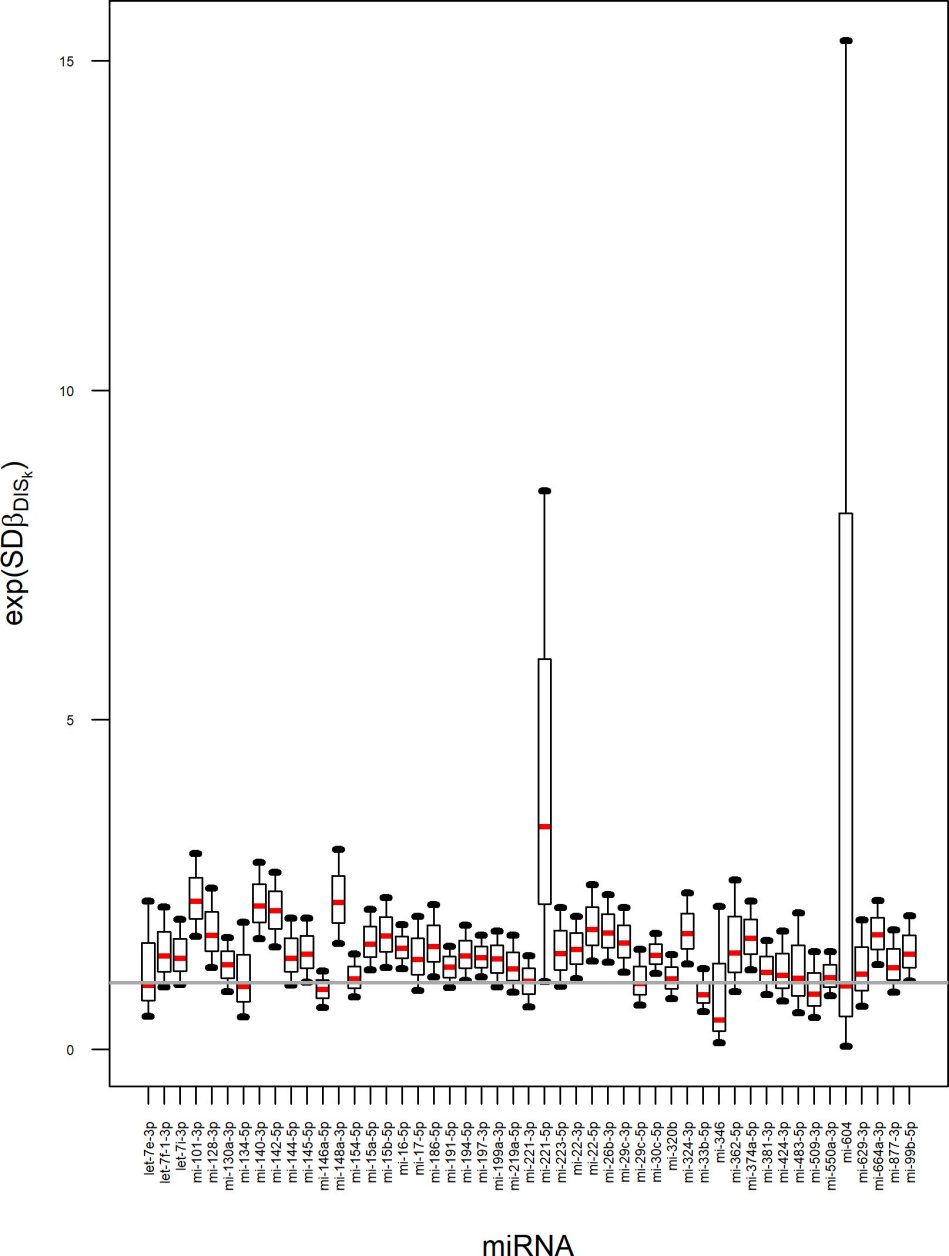
The summary of a marginal posterior distribution representing fold change between disease and control subjects for 49 miRNA. The distribution was summarized as a boxplot with 5th, 25th, 50th, 75th and 95th percentile. Grey line denotes no effect for miRNAs.

Developing the multilevel model we evaluated the effect of health status on miRNA levels (via fold change) by plotting exp(*β*_*DIS*,*k*_) and associated uncertainty for each miRNA (Fig 2). The miRNA levels are generally higher in patients than in healthy individuals with different level of uncertainty. The uncertainty is higher for miRNAs with larger number of missing measurements. The miRNAs for which 90% credible interval is above or below the grey horizontal line could be claimed to be associated with the disease (their levels differ between patients and controls) assuming the model and the available data. As an example the fold changes (median (5^th^-95^th^ percentiles) of exp(*SD*∙*β*_*DIS*,4_) = 2.24 (1.7–2.96)), exp(*SD*∙*β*_DIS,9_) = 2.11 (1.54–2.68)) and exp(*SD*∙*β*_*DIS*,13_) = 2.22 (1.59–3.03)) were determined for miR-101-3p, miR-142-5p and miR-148a-3p. The above-mentioned miRNAs were characterized by quite low percentage of missing data i.e. 4.49%, 0% and 26.4%. On the other hand, those miRNA with high proportion of missingness, i.e. miR-221-5p and miR-604 (94.38% and 98.88%) provide very uncertain predictions (their credible interval is consistent with a large range of possible fold changes). To decrease this uncertainty, more data for these miRNAs should be gathered.

### Usefulness of miRNAs in cancer detection

**Fig 3:**
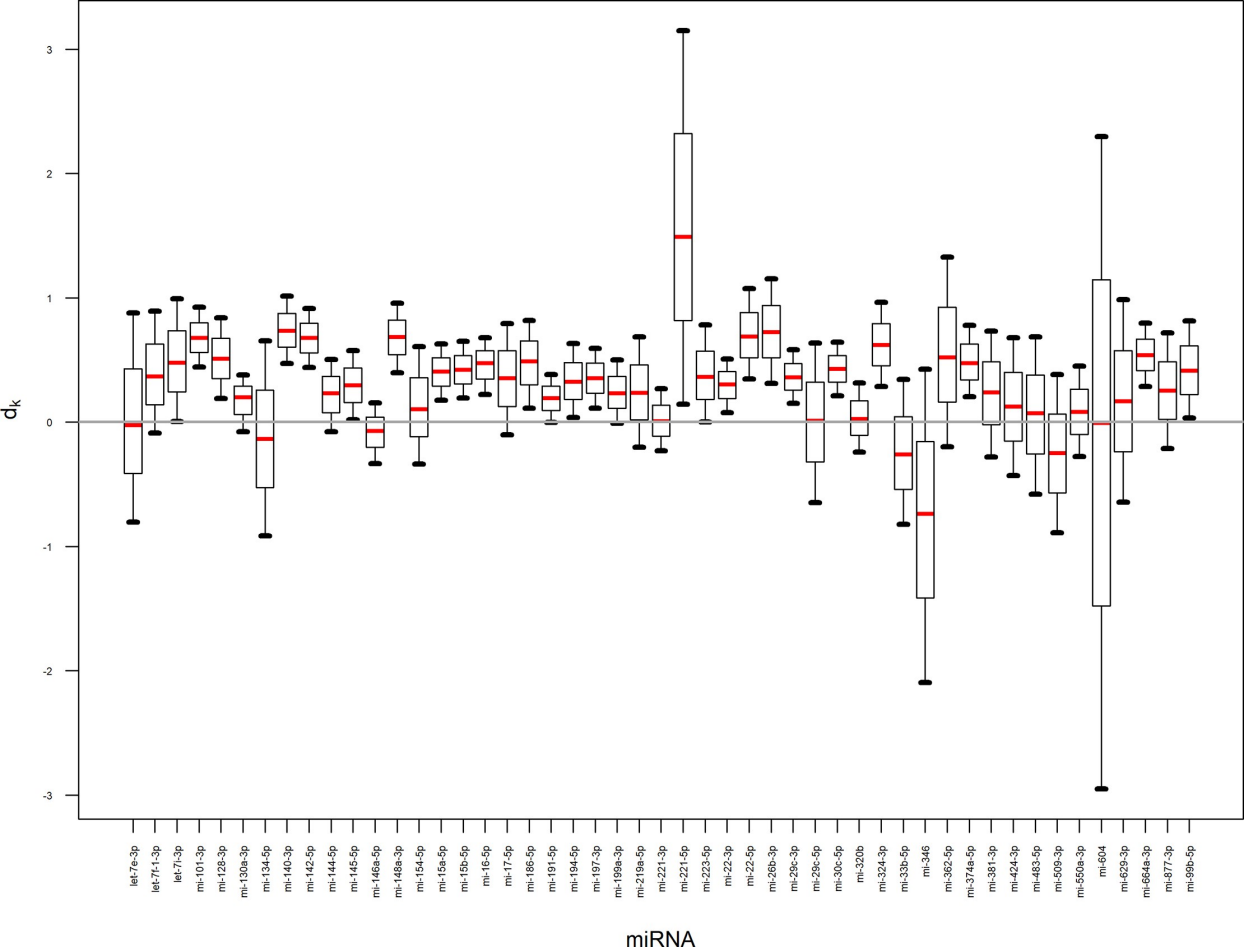
The summary of a marginal posterior distribution of an effect size for 49 miRNA. The distribution was summarized as a boxplot with 5th, 25th, 50th, 75th and 95th percentile. Grey line describes no effect.

By simulations (rather than simply point estimates of parameters), the inferential uncertainty can be propagated into other interesting quantities, like effect size. In this work we estimated the effect size to discuss the difference in miRNA levels between healthy and control subjected on the scale of data variability (Fig 3). The larger the difference the more promising the miRNA for the purpose of diagnosis (to calculate probability of disease). The effect sizes for miRNAs that are characterized by large negative (miR-346) or positive (miR-221-5p) values indicate that for those miRNA it is worth to do more experiments to fully confirm their usefulness in diagnosis. On the other hand if one is willing to select one miRNA for diagnosis based on this data only, three miRNAs (miR-101-3p, miR-142-5p and miR-148a-3p) with effect sizes that are far away from zero would be a good choice as they have the greatest probability of having small effect size. Since they are highly correlated (> 0.95), they carry essentially the same information about health status of the patients.

To calculate the AUC under the ROC curve and further evaluate which combination of miRNAs has the greatest discrimination ability we used 10-fold cross-validation. The one-miRNA-at-a-time AUC under the ROC curve are presented in Table A in S1 Appendix. For the mentioned three miRNAs, i.e miR-101-3p, miR-142-5p and miR-148a-3p, AUC was estimated at 0.65 (0.64–0.66), 0.65 (0.64–0.67), 0.65 (0.62–0.67) suggesting their limited discrimination potency. There is a limited benefit in using more than one miRNA for discrimination, i.e. the use of three miRNAs together led to a very similar AUC of 0.65 (0.63–0.67). There is also a little benefit in adding other miRNAs (i.e. miRNA with missing values being less than 10%). For this subset, the AUC increased to 0.72 (0.69–0.75). This small increase is a consequence of a high correlation between miRNA levels measured in the study.

### Model evaluation and estimation of model parameters

**Fig 4:**
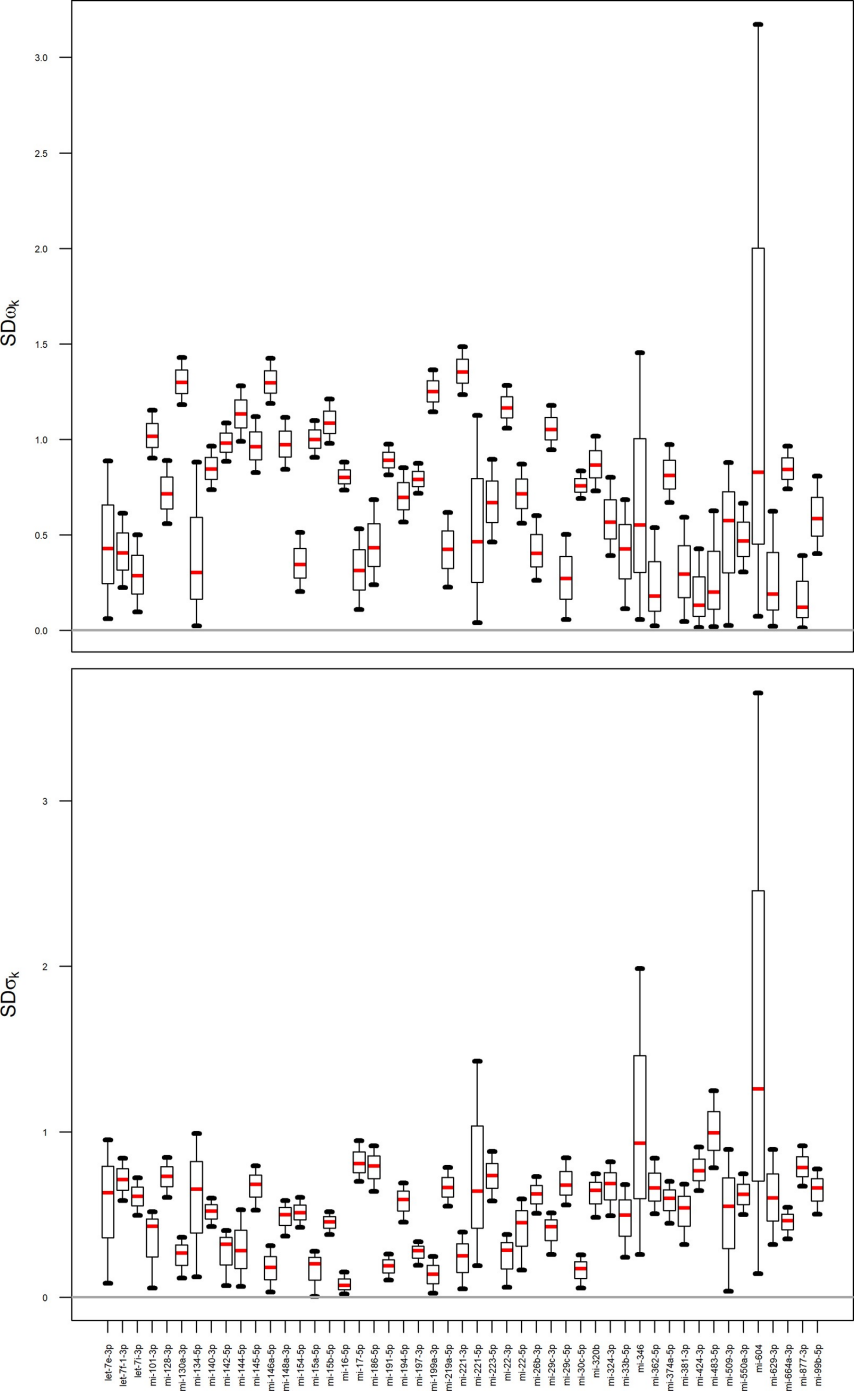
The summary of a marginal posterior distribution of *σ*_*j*,_ *ω*_*j*,_ parameters for 49 miRNA summarized as boxplots with 5^th^, 25^th^, 50^th^, 75^th^, 95^th^ percentiles. A large uncertainty was observed for those miRNA for which missing values were reported in high proportion.

The plot of weighted residuals and weighted residuals versus fitted values (Fig A in S1 Appendix) indicated that the variability of the observations were rather constant across miRNAs, with fairly similar spreads at the fitted values. We therefore conclude no bias or trend in model prediction and therefore conclude good specification of the model. The summary of posterior distributions for standard deviations of assay and inter-individual variability *σ*_*k*_, *ω*_*k*_ for each miRNA were demonstrated in Fig 4.

### False Discovery Rate *p* values

Four miRNA levels were found to be significantly different between patients and controls i.e. miR-101-3p, miR-140-3p, miR-142-5p, miR-148a-3p based on FDR *p*-values. The corresponding effect size (*d*_*FDR*_) for these miRNA was 0.63, 0.64, 0.6, 0.56. We also observed that two miRNAs which were not significant (according to null hypothesis significance testing) after FDR correction were characterized by the *d*_*FDR*_ > 1 i.e. miR-221-5p (*d*_*FDR*_ = 1.77) and for miR-346 (*d*_*FDR*_ = 1.43). The *d*_*FDR*_ > 1 without significance after FDR correction could be a consequence of high proportion of missingness for these miRNAs (94.38% and 95.51%). The four miRNAs which were significantly different between patients and controls were also characterized by a relatively low percentage of missingness (Table A in S1 Appendix).

## Discussion

Unlike computational–based models aimed at finding a candidate miRNA potentially associated with the disease accompanied by further confirmation its relevance in experiments, the general idea of this work was to judge on the practical relevance of miRNAs in the presence of measurement noise and data variability by proposing the data generating process.

Specifically, we firstly investigated the relationship between the presence of ovarian cancer and miRNA expression via multilevel Bayesian model. Secondly, we assessed the effect of disease on miRNA levels controlling for differences in covariates and modelling the covariance matrix. The effect size and uncertainties around its estimates allowed to judge how certain the magnitude of miRNAs levels can be estimated which constituted an aid in terms of interpretation of the practical relevance of 49 miRNAs measurements for diagnostic purposes.

Under the partial-pooling scenario served by the multilevel Bayesian framework, the parameters’ estimates were pulled toward the population mean with a standard deviation set to unity (weekly-informative prior), leading to a reduction of false–discoveries. Another aspect of partial pooling is related to information sharing allowing to estimate individual model parameters. In other words, using multilevel modeling we can make inference on each miRNA borrowing information from other miRNAs.

In the context of gene expression study, there are many factors that affect the obtained results e.g. missing values identified during data generating process evolving mainly from detection sensitivity, contamination, error induced in experimental operations or inappropriate data pre-processing. Their occurrence is unavoidable leading to uncertainty of model estimates and affecting final conclusions if not properly accounted for [43]. For studies considering differentially expressed genes, estimation of a fold–change is the simplest and still widely used measure to identify gene-specific changes, however rarely with a measure of its reliability (due to assumption that all genes exhibit the same level of noise). In the literature, a “significance analysis of microarrays” (SAM) is a common and simple approach utilizing genome–wide information to account for a signal-to-noise ratio [44].

Apart from the existence of this measure and discussing the difference in miRNA levels estimated on the scale of data variability, we used the effect size with associated uncertainty to judge on the relevance of measuring miRNA levels. As evidenced by *a posteriori* distribution of effect sizes of individual miRNA among patients in relation to the control group, several effect sizes are possibly large (above 1) due to large uncertainty. Under such scenario, one should always keep in mind that the true value may lie between those bounds and to reduce this uncertainty around estimates, more data is required. The distribution of effect sizes were also characterized by a negative values which indicate that for some miRNA a decrease in cancer patients is plausible [45].

The posterior distribution of effect size and uncertainties around its estimates allowed for a more sophisticated and intuitive judgment on health status effect exerted on miRNA levels. In our opinion, probabilistic assessments of uncertainty around model parameters and predictions is more convincing especially when making decision on the validity of miRNA measurements for cancer detection [46,47].

In this study we showed that the effect size along with the associated uncertainty can be a useful measure to assess the potential diagnostic value of each miRNA. Accompanying credible intervals for the AUC constituted a kind of “double-check” of miRNAs’ diagnostic value. However to prove usefulness for diagnostic purposes, more studies are needed to quantify the added predictive value of individual miRNAs’ measurements.

Unlike Bayesian concept to potential markers discovery, under Frequentists approach the effect size is treated as a fixed value addressing the question how much the data differ ‘significantly’ from that expected under the null. The vast majority of research uses the Frequentists concept completely ignoring the probability of raised hypothesis [48] and reporting only those results which are statistically significant. Usually, when replicating the original case-control study under the same conditions or under conditions as much similar to the original study as possible, researchers obtain negative results (zero or small effect). This is commonly explained via the presence of a wide sample-to-sample variability in the data, small sample size etc. [49,50]. For this reason, the same experiment will probably result in a substantially different *p*-value which questions reliability of results generally obtained in those experiments, even with high statistical power of a test [51,52].

In observational studies aimed at selecting potential disease indicators, we can observe a high probability of effects’ overestimation, high false negative rate resulting from study design and sample size (often inadequate in terms of complexity of the task), lack of confounders adjustment and ignorance of correlations between studied features. The above-mentioned factors influence this one-at-a-time feature selection to a great extent leading to poor predictive performance of developed models and thus generating non-reproducible results. Given that biology is complex and variability in the data is always present, for data analysis purposes we should apply methods that fit better the data i.e. those based on penalization, like the proposed multilevel model [53,54].

Liu et al. however, [55] discuss whether multilevel models may outperform single-gene-at-a-time analysis or SAM in genomics studies. The authors point out that multilevel models can have good performance in case of variance stabilization, however differential expression can be more reasonably analysed with poisson and negative binomial models. Moreover, they underlie that research objective is a key when it comes to decision on the analysis method used. If the goal is gene selection, more computing intensive shrinkage approach should be considered. If we expect large signals changes, fold changes and tail probabilities appear to be the best statistics, otherwise when estimating reliably measured differential expression, the signal-to-noise ratios and Bayes factors is a good choice.

In this study, we built a data-driven model describing the effect of disease and associated covariates on miRNA level simultaneously accounting for the presence of variability. We modelled miRNA data using the normal distribution (with between-subject variability modelled via multivariate normal distribution). Under this scenario, the use of poisson or negative binomial distribution to model miRNA data was unnecessary and could only complicate the model. Although the use of easy-to-use SAM could be a more simple approach to practitioners, we could not apply this measure in this study as we observed significant age difference between patients and healthy individuals (which may be considered a limitation of the study). The use of multilevel model with age and bodyweight adjustment allowed to take the potentially confounding effects of age and body weight into account. The lack of endogenous controls (housekeeping gene) for quantitative control and normalization may also be considered as an limitation of the study.

## Conclusions

The relevance of the most promising miRNAs for cancer diagnosis identified in this work (miR-101-3p, miR-142-5p, miR-148a-3p) is rather limited. There are however several miRNAs for which the inferences are uncertain. When analyzing such small data from an observational study caution is always needed as the effects could be biased due to presence of unaccounted confounders. The proposed approach should be considered a more natural statistical formalization of the scientific process of evaluating the evidence. The Bayesian posterior quantifies the uncertainty about the model parameters allowing to make various decision, i.e. assess the usefulness of miRNA for cancer detection.

## Supporting information

S1 Data Raw data. (CSV)

S1 Appendix Experimental procedures, codes for Bayesian multilevel model and supporting figure and table. (DOCX)
