# Supplementary material for: Bayesian multilevel model of micro RNA levels in ovarian-cancer and healthy subjects
Source: PLoS One. 2019 Aug 29;14(8):e0221764. doi: 10.1371/journal.pone.0221764 (PMC6715278; doi:10.1371/journal.pone.0221764)
Supplement: S1 Appendix — (DOCX) [file pone.0221764.s002.docx]

**PLoS One Supporting Information Appendix S1**

**Article title: Bayesian multilevel model of micro RNA levels in ovarian-cancer and healthy subjects**

Authors: Paweł Wiczling, Emilia Daghir-Wojtkowiak, Roman Kaliszan, Michał Jan Markuszewski, Janusz Limon, Magdalena Koczkowska, Maciej Stukan, Alina Kuźniacka, Magdalena Ratajska

The following Supporting Information is available for this article

# S1 Text. Experimental procedure.

**S1 Text. Codes for multilevel Bayesian modeling.**

**S1 Table A. The FDR adjusted *p*-value, percent of missing data, one-miRNA-at-a-time AUC under the ROC curve.**

**S1 Fig. A. Goodness of fit plots.**

# S1 Text. Experimental procedure

## The sample quality control

Since reproducible RNA isolation can be difficult from certain types of samples, known RNA spike-ins were added to the sample prior to RNA extraction. The quality of RNA was checked by UniSp2, UniSp4 and UniSp5, while the RT- reaction control was performed using UniSp3 and UniSp6. After conducting the RT-qPCR, wells detecting the RNA spike-ins were identified and compared. The results confirmed that both reverse transcription and qPCR were successful and that none of the samples, used in the experiments, contained any inhibitors.

The level of hemolysis was assessed by examining miRNA-451 that is expressed in red blood cells and is relatively stable in serum and plasma, and miRNA-23a that is not affected by hemolysis. Samples quality was addressed by calculating the dCp value of miRNA-23a and miRNA-451with the cutoff ~7. The dCp above seven indicated a high risk of hemolysis; therefore, samples with dCp>7 were excluded from further analysis.

Additionally, in each RT-qPCR reaction, no-template control was included and profiled the same way as other samples.

## Workflow of miRNAs expression profiling

4μL RNA were reverse transcribed in 20μL reactions using the miRCURY LNA™ Universal RT microRNA PCR, Polyadenylation and cDNA synthesis kit (Exiqon Inc., Woburn, MA). cDNA was diluted 50x and assayed in 10μL PCR reactions according to the protocol for miRCURY LNA™ Universal RT microRNA PCR. Each microRNA was assayed twice by qPCR on the microRNA Ready-to-Use PCR, Custom Pick and Mix Panel using ExiLENT SYBR® Green master mix. The amplification was performed in a LightCycler® 480 Real-Time PCR System (Roche) in 384 well plates. The amplification curves were analyzed using the Roche LC software, both for determination of the threshold cycle (Ct) (by the 2nd derivative method) and for melting curve analysis (Exiqon Inc.). As a result of real-time PCR panel profiling of 49 miRNA in 178 individuals (118 controls and 59 patients) in two replicate reactions, raw CT values were determined and further used to retrieve data generation process, account for missing values and evaluate sample-to-sample variation among replicate reactions.

**S1 Text. Codes for multilevel Bayesian modeling**

# In the description of methodology we use standard deviation while the codes applied in JAGS use #precision model

{

for(i in 1:I){

sETA [i,1:K] ~ dmnorm(vzeros[1:K], Omega.inv[1:K, 1:K])

}

for(i in 1:I){

for(k in 1:K){

ETA[i,k] <- xi[k]*sETA[i,k]

}}

for(n in 1:N){

logC[i] <- miu[MIRNAID[n]] + ETA[ID[n], MIRNAID[n]] + betaDIS[MIRNAID[n]]*DIS[ID[n]] + betaAGE[MIRNAID[n]]*AGE[ID[n]] + betaBW[MIRNAID[n]]*BW[ID[n]]

Y[n] ~ dnorm(logC[n], tausigma[MIRNAID[n]])

Ycond[n] ~ dnorm(logC[n], tausigma[MIRNAID[n]])

}

# missing values for DIS, AGE, BW

for (i in 1:I) {

DIS[i]~dbern(0.3333)

AGE[i] ~ dnorm(0,1)

BW[i] ~ dnorm(0,1)

}

# priors

Omega.inv[1:K, 1:K] ~ dwish(Omegainvprior[1:K, 1:K], df)

somega[1:K, 1:K] <- inverse(Omega.inv[1:K, 1:K])

for(k in 1:K){

for (k.prime in 1:K){

rho[k,k.prime] <- somega [k,k.prime]/sqrt(somega [k,k]* somega[k.prime,k.prime])

}

omega[k] <- abs(xi[k])*sqrt(somega[k,k]) # scaled SD of parameter k

}

df <- K + 1

for (k in 1:K ) {

miu[k] ~ dnorm(0, 0.04) # 1/5^2

sigma[k] ~ dnorm(0, 1)T(0,)

tausigma[k] <- 1/(sigma[k]*sigma[k])

xi[k] ~ dnorm(0, 1)T(0,)

betaDIS[k] ~ dnorm(0, 1)

betaAGE[k] ~ dnorm(0, 1)

betaBW[k] ~ dnorm(0, 1)

d[k] <- betaDIS[k]/pow(sigma[k]^2+omega[k]^2,0.5)

}

}

**S1 Table A.** Benjamini & Hochberg adjusted *p-*values for each miRNA comparing the levels of miRNA in case and control group along with percentage of missingness for each miRNA and one-miRNA-at-a-time AUC under the ROC curve from 10-fold cross-validation.

| No | | miRNA | FDR adjusted *p*-value | Percent of missing data | AUC (5th-95th credible interval) |
| --- | --- | --- | --- | --- | --- |
| 1 | | hsa-let-7e-3p | 0.561211 | 87.08% | 0.49 (0.41-0.58) |
| 2 | | hsa-let-7f-1-3p | 0.954784 | 62.36% | 0.59 (0.52-0.65) |
| 3 | | hsa-let-7i-3p | 0.762538 | 57.30% | 0.50 (0.43-0.58) |
| 4 | | hsa-miR-101-3p | **0.000275** | 4.49% | 0.65 (0.64-0.66) |
| 5 | | hsa-miR-128-3p | 0.050618 | 28.65% | 0.62 (0.58-0.66) |
| 6 | | hsa-miR-130a-3p | 0.561211 | 1.12% | 0.52 (0.47-0.56) |
| 7 | | hsa-miR-134-5p | 0.515118 | 89.33% | 0.51 (0.42-0.62) |
| 8 | | hsa-miR-140-3p | **0.004534** | 3.37% | 0.62 (0.60-0.64) |
| 9 | hsa-miR-142-5p | | **0.000275** | 0.00% | 0.65 (0.64-0.67) |
| 10 | hsa-miR-144-5p | | 0.978476 | 9.55% | 0.50 (0.47-0.54) |
| 11 | hsa-miR-145-5p | | 0.232225 | 2.81% | 0.58 (0.54-0.60) |
| 12 | hsa-miR-146a-5p | | 0.503384 | 0.00% | 0.47 (0.39-0.56) |
| 13 | hsa--miR-148a-3p | | **0.007945** | 26.40% | 0.65 (0.62-0.67) |
| 14 | hsa-miR-154-5p | | 0.561211 | 59.55% | 0.50 (0.43-0.58) |
| 15 | hsa-miR-15a-5p | | 0.050618 | 0.00% | 0.57 (0.54-0.59) |
| 16 | hsa-miR-15b-5p | | 0.103447 | 1.69% | 0.55 (0.53-0.58) |
| 17 | hsa-miR-16-5p | | 0.050618 | 0.56% | 0.57 (0.55-0.58) |
| 18 | hsa-miR-17-5p | | 0.922750 | 56.18% | 0.52 (0.46-0.59) |
| 19 | hsa-miR-186-5p | | 0.834826 | 38.20% | 0.54 (0.48-0.59) |
| 20 | hsa-miR-191-5p | | 0.380797 | 0.00% | 0.53 (0.48-0.57) |
| 21 | hsa-miR-194-5p | | 0.167046 | 30.34% | 0.58 (0.55-0.62) |
| 22 | hsa-miR-197-3p | | 0.098285 | 0.00% | 0.58 (0.56-0.59) |
| 23 | hsa-miR-199a-3p | | 0.563933 | 2.25% | 0.51 (0.46-0.54) |
| 24 | hsa-miR-219a-5p | | 0.561211 | 60.67% | 0.56 (0.50-0.63) |
| 25 | hsa-miR-221-3p | | 0.820053 | 1.12% | 0.45 (0.38-0.55) |
| 26 | hsa-miR-221-5p | | 0.380797 | 94.38% | 0.52 (0.37-0.70) |
| 27 | hsa-miR-223-5p | | 0.550557 | 56.18% | 0.60 (0.54-0.66) |
| 28 | hsa-miR-22-3p | | 0.350210 | 0.00% | 0.53 (0.48-0.56) |
| 29 | hsa-miR-22-5p | | 0.293669 | 44.38% | 0.58 (0.52-0.63) |
| 30 | hsa-miR-26b-3p | | 0.191345 | 51.12% | 0.61 (0.56-0.67) |
| 31 | hsa-miR-29c-3p | | 0.289899 | 3.37% | 0.54 (0.52-0.56) |
| 32 | hsa-miR-29c-5p | | 0.380797 | 73.60% | 0.47 (0.38-0.56) |
| 33 | hsa-miR-30c-5p | | 0.183319 | 1.12% | 0.57 (0.56-0.59) |
| 34 | hsa-miR-320b | | 0.762538 | 5.62% | 0.43 (0.36-0.55) |
| 35 | hsa-miR-324-3p | | 0.098285 | 32.58% | 0.60 (0.55-0.65) |
| 36 | hsa-miR-33b-5p | | 0.100521 | 76.97% | 0.50 (0.42-0.58) |
| 37 | hsa-miR-346 | | 0.503384 | 95.51% | 0.51 (0.41-0.61) |
| 38 | hsa-miR-362-5p | | 0.954784 | 78.09% | 0.53 (0.43-0.62) |
| 39 | hsa-miR-374a-5p | | 0.289899 | 33.71% | 0.60 (0.57-0.63) |
| 40 | hsa-miR-381-3p | | 0.503384 | 67.42% | 0.49 (0.41-0.57) |
| 41 | hsa-miR-424-3p | | 0.820053 | 69.66% | 0.49 (0.41-0.57) |
| 42 | hsa-miR-483-5p | | 0.525155 | 74.72% | 0.60 (0.50-0.69) |
| 43 | hsa-miR-509-3p | | 0.183319 | 71.91% | 0.50 (0.43-0.58) |
| 44 | hsa-miR-550a-3p | | 0.490379 | 48.31% | 0.50 (0.44-0.56) |
| 45 | hsa-miR-604 | | NA | 98.88% | 0.50 (0.40-0.62) |
| 46 | hsa-miR-629-3p | | 0.812545 | 89.89% | 0.50 (0.41-0.58) |
| 47 | hsa-miR-664a-3p | | 0.050618 | 7.87% | 0.60 (0.58-0.62) |
| 48 | hsa-miR-877-3p | | 0.561211 | 58.99% | 0.57 (0.48-0.65) |
| 49 | hsa-miR-99b-5p | | 0.537474 | 48.88% | 0.58 (0.52-0.64) |

# Supplementary figure


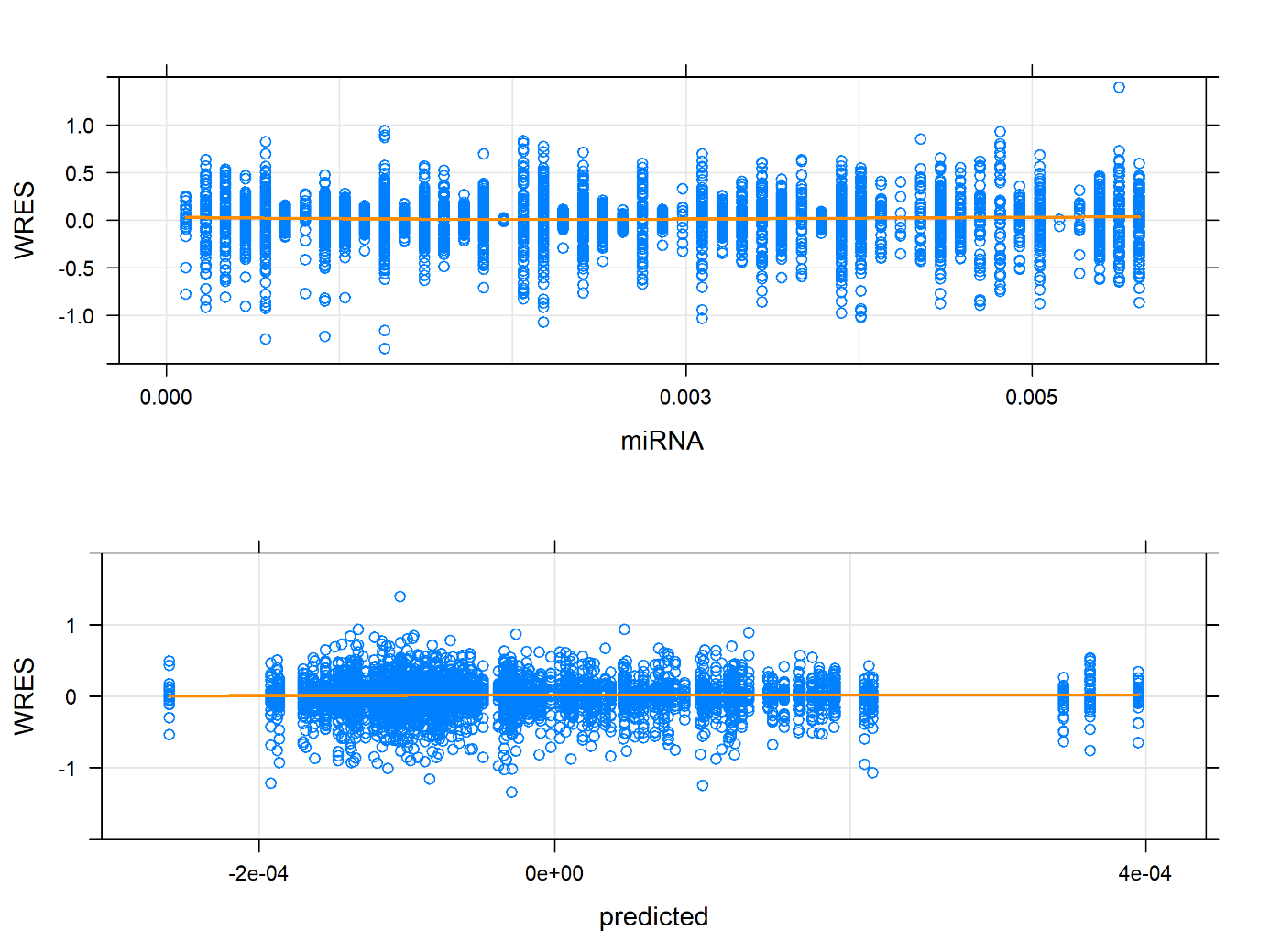


**Fig. A.** Goodness of fit plots. Weighted residuals (top) and weighted residuals versus fitted values (bottom) for 49 miRNAs. At the top figure the dots are distributed across the line of identity. At the bottom figure the dots are quite evenly distributed across zero with no visible pattern or trend. Both graphs suggest good specification of the model.
